# A validation study of microscopy versus quantitative PCR for measuring *Plasmodium falciparum* parasitemia

**DOI:** 10.1186/s41182-019-0176-3

**Published:** 2019-08-27

**Authors:** Emma Ballard, Claire Y. T. Wang, Tran Tinh Hien, Nguyen Thanh Tong, Louise Marquart, Zuleima Pava, Joel Tarning, Peter O’Rourke, James S. McCarthy

**Affiliations:** 1 QIMR Berghofer Medical Research Institute, Brisbane, Australia; 2 Queensland Paediatric Infectious Diseases Laboratory, Centre for Children’s Health Research, Brisbane, Australia; 3 Oxford University Clinical Research Unit—Hospital for Tropical Diseases, Ho Chi Minh City, Vietnam; 4 Mahidol-Oxford Tropical Medicine Research Unit, Faculty of Tropical Medicine, Mahidol University, Bangkok, Thailand; 5 Centre for Tropical Medicine and Global Health, Nuffield Department of Medicine, Oxford University, Oxford, UK; 6 The University of Queensland, Brisbane, Australia

**Keywords:** Microscopy, qPCR, *Plasmodium falciparum*, Validation

## Abstract

Microscopy and 18S qPCR are the most common and field-friendly methods for quantifying malaria parasite density, and it is important that these methods can be interpreted as giving equivalent results. We compared results of quantitative measurement of *Plasmodium falciparum* parasitemia by microscopy and by 18S qPCR in a phase 2a study. Microscopy positive samples (*n* = 355; median 810 parasites/μL [IQR 40–10,471]) showed close agreement with 18S qPCR in mean log_10_/mL transformed parasitemia values by paired *t* test (difference 0.04, 95%CI − 0.01–0.10, *p* = 0.088). Excellent intraclass correlation (0.97) and no evidence of systematic or proportional differences by Passing–Bablok regression were observed. 18S qPCR appears to give equivalent parasitemia values to microscopy, which indicates 18S qPCR is an appropriate alternative method to quantify parasitemia in clinical trials.

## Background

Microscopy is the gold standard method for identifying and quantifying malaria parasites [1]. Microscopists calculate parasitemia (parasites/microliter (μL) of whole blood) as the parasites counted within a given number of microscope fields divided by the number of WBCs (thick film) or RBCs (thin film) in μL of whole blood multiplied by the total WBC (thick film) or RBC (thin film) counted within those fields [2]. The microscopy detection limit is generally 11–50 parasites/μL [3].

Nucleic acid amplification testing (NAAT) methods detect and quantify parasitemia with greater sensitivity and precision than microscopy, particularly for low levels of parasitemia [4]. Quantitative NAAT methods include quantitative PCR (qPCR) and quantitative reverse transcriptase PCR (qRT-PCR). NAAT methods for malaria target either specific DNA sequences of the parasite’s genome, such as the gene encoding the 18S ribosomal RNA gene (18S rDNA), or a constitutively transcribed RNA sequence such as 18S rRNA of asexual parasites [1].

The World Health Organization (WHO) has published standardised protocols for microscopy [5, 6]. However, no equivalent protocols exist for NAAT. The Minimum Information for Publication of Quantitative Real-Time PCR Experiments (MIQE) guidelines [7] have led towards a more standardised approach to malaria NAAT [8, 9]. Murphy et al. [4] established the first formal external quality assurance (EQA) program to promote the reliability and comparability of results for NAAT across centres undertaking volunteer infection studies (VIS). In 2018, WHO published an operational manual for an ongoing EQA program for malaria NAAT [10].

Our aim was to compare microscopy and qPCR parasitemia values to demonstrate that qPCR is a suitable alternative to microscopy for quantifying parasitemia in clinical trials.

## Methods

Participants with uncomplicated *Plasmodium falciparum* malaria were treated with a single dose of cipargamin (KAE609) in an open-label phase 2a study in Vietnam, as described by Hien et al. [11]. Twenty-five adult males had 6 mL of blood taken 4–6 hourly until they had two consecutive negative blood films, and subsequently once daily until day 8.

Experienced microscopists followed standard procedures to count asexual parasites using both thick and thin blood films shortly after blood collection [6]. First, 200 WBCs were counted on thick films. If the parasite count was less than 10 after 200 WBCs, then up to 500 WBCs were counted. Thin films were used where the number of parasites exceeded 250 per 50 WBCs.

For the 18S qPCR, DNA was extracted from a 200-μL volume of packed RBCs using the QIAamp DNA blood mini kit (Qiagen). The *Plasmodium*-specific qPCR was performed on-site in real time using the protocol described by Imwong et al. [9] with primers and probes developed by Kamau et al. [12]. The method uses a hydrolysis probe real-time qPCR targeting the 18S rRNA gene with a detection limit of 22 parasites/mL (SD 5). Samples were run in triplicate. Each 18S qPCR run contained a positive and negative water control and internal phocine herpesvirus control. Cycle threshold values greater than 50 were considered non-detectable.

To account for possible confounding of results by circulating gametocytes, RNA was extracted from frozen packed RBCs using the QIAamp DNA blood mini kit (Qiagen) with DNase treatment and tested for the presence of the *P. falciparum* female gametocyte-specific mRNA *pfs25*, using the primers, probe, and protocol described by Pasay et al. [13]. The ratio of log_10_ gametocytes/mL (*pfs25* qRT-PCR) to log_10_ parasites/mL (18S qPCR) was then calculated for the 20 samples with both gametocyte and asexual parasite counts.

Log_10_ parasitemia values using microscopy and 18S qPCR were summarised by mean, standard deviation, coefficient of variation, median, interquartile range, and range. We used paired *t* test, intraclass correlation coefficient (ICC), and Passing-Bablok regression to assess for agreement between log_10_ parasitemia values. ICC for consistency was calculated using a mixed effects model without interaction. The Passing-Bablok regression 95% confidence interval (CI) was calculated with the bootstrap (quantile) method. Regression and residual plots were examined. The reference method was microscopy. All analyses were conducted in R Studio (version 1.0.136, R version 3.2.5).

## Results

Of the 740 blood samples collected, 268 microscopy results were reported as non-detectable, 92 samples were clotted and unsuitable for 18S qPCR, and 23 were missing values. Two 18S qPCR assay results failed quality control checks, resulting in 355 matched samples across 22 participants. Median parasitemia value by microscopy was 810 parasites/μL (IQR 40–10,471).

Log_10_ parasitemia values from microscopy were marginally lower (mean 5.81 log_10_ units/mL) than values from 18S qPCR (mean 5.86 log_10_ units/mL) (Table 1). Standard deviations and coefficients of variation were similar between methods. All samples with parasites detected by microscopy were also positive by 18S qPCR.

**Table 1:** Comparison of log_10_ parasitemia values/mL by microscopy and 18S qPCR

	Microscopy	18S qPCR
*n*	355	355
Mean (SD)	5.81 (1.31)	5.86 (1.33)
CV%	22.5	22.8
Median (IQR)	5.91 (4.60–7.02)	6.00 (4.65–7.00)
Range	3.48–7.88	2.30–8.18

*CV%* coefficient of variation, *IQR* interquartile range, *SD* standard deviation

**Fig. 1:**
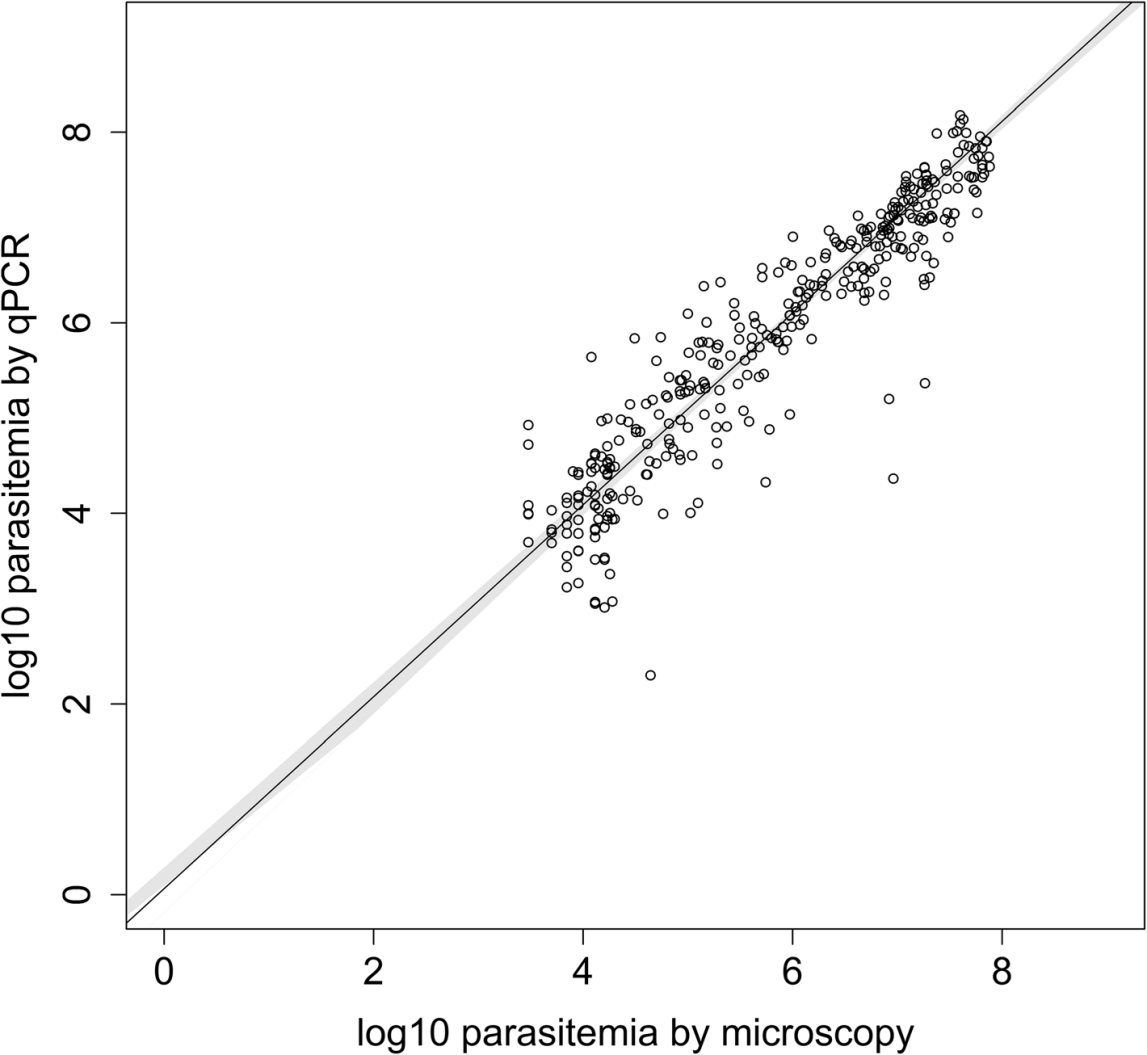
Passing–Bablok regression fit comparing microscopy and 18S qPCR. The plot shows log_10_ parasitemia by microscopy and 18S qPCR. The solid black line represents the fitted Passing–Bablok regression line. The 95% confidence bounds, in grey, were calculated using the bootstrap quantile method

The high ICC value indicated excellent consistency between microscopy and 18S qPCR (Table 2). The paired *t* test indicated that log_10_ parasitemia values/mL were not significantly different between microscopy and 18S qPCR (mean difference 0.04 [95% CI − 0.01–0.10], *p* = 0.088). There was no evidence of a systematic or proportional bias by Passing–Bablok regression but a slight tendency towards non-linearity at lower levels of parasitemia (H statistic: 1.50; *p* < 0.05) (Fig. 1).

**Table 2:** Agreement statistics for log_10_ parasitemia/mL by microscopy and 18S qPCR

	Microscopy versus 18S qPCR
*n*	355
ICC (95% CI)	0.965 (0.956–0.971)
Paired *t* test (95% CI)	
Mean difference (95% CI)	0.04 (− 0.01–0.10)
*p* value	0.088
Passing–Bablok regression	
Intercept (95% CI)	0.06 (− 0.18–0.28)
Slope (95% CI)	1.01 (0.97–1.04)
H statistic	1.50

*CI* confidence interval, *ICC* intraclass correlation coefficient

Our 18S qPCR assay was developed for clinical trials where the upper limit of quantification (ULoQ) is 250,000 parasites/mL [9]. Half of the samples tested had parasitemia values above this ULoQ. We conducted sensitivity analysis to examine whether extrapolation of 18S qPCR values above the ULoQ was reasonable (*n* = 155 ≤ 250,000 parasites/mL, mean difference 0.07 log_10_ units/mL [95% CI − 0.01–0.15, *p* = 0.10]), and found our conclusions unchanged, which indicates that extrapolation beyond the reference range was valid.

Across matched samples analysed by both18S qPCR and *pfs25* qRT-PCR assay the ratio of gametocytes to total parasites averaged 0.006 (SD 0.010) and ranged between 0.000 and 0.050. Thus, our comparison of microscopy with 18S qPCR was not confounded by the presence of gametocytes.

## Discussion

Our findings support the use of the 18S qPCR assay in clinical trials to quantify a wide range of parasitemia values, based on a strong agreement with microscopy. This has application to clinical trials in endemic settings (such as [11]) and to VIS in non-endemic settings.

Parasitemia values from microscopy and from 18S qPCR showed good agreement and no evidence of systematic or proportional bias by Passing–Bablok regression, a method commonly used in clinical laboratory settings [14]. Our findings of a mean difference of 0.04 log_10_ units/mL are supported by several smaller studies. Nwakanma et al. [15] calculated a mean difference of − 0.5 log_10_ units (95% CI − 1.4–0.4) for 81 thick films with values from an 18S rRNA gene-targeted qPCR. Kamau et al. [16] reported a much larger mean difference of − 0.87 log_10_ units for 60 samples, although they used averaged thick or thin film counts using a multiplex qPCR containing an 18S rRNA gene target.

Although microscopy is the gold standard for quantifying parasitemia, microscopy cannot quantify parasitemia at low levels and its accuracy is influenced by the film type (thick or thin), microscopist’s expertise, and WBC count (theoretical or actual) used [2]. Quantification of sexual lifecycle stages may not be included in microscopy counts, such as in this study. However, we did not find evidence of over-estimation by 18S qPCR due to low numbers of gametocytes in matched samples. If deconvolution of asexual parasitemia versus gametocytemia is required, then inclusion of qRT-PCR assays targeting specific life cycle stages is recommended [13].

18S qPCR assays cannot discern whether target DNA is within a viable or dead parasite, or elsewhere, such as cell-free DNA. Parasite counts from 18S qPCR assays could be influenced by cell-free *Plasmodium* DNA in the saliva, blood, or stool of malaria-infected humans [17]. This could lead to overestimation of intact and viable parasites if parasites from target DNA elsewhere are counted [18]. However, the agreement between the parasitemia values estimated by microscopy and 18S qPCR in our study (patients treated with cipargamin) suggests confounding due to cell-free DNA is unlikely.

## Conclusions

In this study, we compared the most common and field-friendly methods for parasite quantification. Although other methods, including flow cytometry [19] and droplet digital PCR [20], can quantify parasitemia without a standard curve, they have high operational costs and are unlikely to be realistic options for many laboratories. In conclusion, our results support the use of 18S qPCR to give equivalent estimates to those from microscopy for *P. falciparum* parasitemia in clinical trials, particularly drug studies where accurate quantification is required across a wide range of parasitemia.

## Data Availability

The datasets analysed during the current study are available from the corresponding author on reasonable request.

## Abbreviations

CI: Confidence interval
EQA: External quality assurance
ICC: Intraclass correlation coefficient
IQR: Interquartile range
MIQE: Minimum Information for Publication of Quantitative Real-Time PCR Experiments
NAAT: Nucleic acid amplification testing
qPCR: Quantitative polymerase chain reaction
qRT-PCR: Quantitative reverse transcriptase polymerase chain reaction
RBC: Red blood cell
SD: Standard deviation
ULoQ: Upper limit of quantification
VIS: Volunteer infection study
WBC: White blood cell
WHO: World Health Organization
